# Pattern identification of lung cancer patients based on body constitution questionnaires (BCQ) and glycoproteomics for precision medicine

**DOI:** 10.1097/MD.0000000000016035

**Published:** 2019-06-14

**Authors:** Wonryeon Cho, Ji Hye Kim, Miseon Jeong, Myeong-Sun Kim, Jinwook Lee, Hyoungwoo Son, Chunhoo Cheon, Sunju Park, Seong-Gyu Ko

**Affiliations:** a Department of Chemistry, Wonkwang University, Iksan, Jeonbuk; b Laboratory of Clinical Biology and Pharmacogenomics, Department of Preventive Medicine, College of Korean Medicine; c Department of Cancer Preventive Material Development, Graduate School, Kyung Hee University; d Department of Korean Medicine, Graduate School of Kyung Hee University; e Department of Preventive Medicine, College of Korean Medicine, Kyung Hee University Dongdaemun-gu, Seoul; f Department of Preventive Medicine, College of Korean Medicine, Daejeon University, Dong-gu, Daejeon, Republic of Korea.

**Keywords:** body constitution questionnaire (BCQ), glycoproteomics, lung cancer, pattern identification, precision medicine, representative sampling, traditional Korean medicine (TKM)

## Abstract

**Background:**

The patient's pattern identification has been used for personalized medicine in traditional Korean medicine (TKM) and aims for patient-specific therapy by Korean medical doctors. The pattern identification in this trial will be diagnosed from body constitution questionnaire (BCQ) with a more objective diagnosis of it but this method still needs a more concrete scientific basis. Glycoproteins are well-known to be associated with diseases (especially cancers) so glycoproteomics can be applied to differentiate pattern identification types of lung cancer patients. Thus, for the first time proteomics approach will be applied to the pattern identification by comparing BCQ assessment in order to establish a scientific basis with clinical proteomics for precision medicine.

**Methods:**

This observational trial will at first diagnose the pattern identification types of lung cancer patients with BCQ assessment and then elucidate their relationships with proteomics. Blood samples will be collected before surgery along with clinical information of participants. The patients’ pattern identification in TKM will be diagnosed from BCQ assessment. Then, lung cancer patients will be divided and pooled into 3 lung cancer entire (LCE) groups according to their pattern identification types (Xu, Stasis, or Gentleness). Three lung cancer representative (LCR) groups will be selected and pooled from each LCE group by selecting those with the same control factors. The 3 LCE groups and the 3 LCR groups from lung cancer patients will be independently analyzed through the glycoproteomics approach based on the patients’ pattern identification. Glycoproteins from the 6 groups will be identified through proteomics approach and then categorized for analysis.

**Discussion:**

This study intends to diagnose pattern identification of patients in TKM with BCQ assessment and proteomics approach. The identification of the glycoproteins in each group will lead to the scientific foundation of personalized medicine in TKM according to patients’ pattern identification for lung cancer therapy. We intend to

(1) diagnose the pattern identification types of lung cancer patients with BCQ under the framework of TKM;

(2) evaluate BCQ assessment with glycoproteomics approach for precision medicine.

**Trial registration:**

ClinicalTrials.gov NCT03384680. Registered 27 December 2017. Retrospectively registered.

## 1 Introduction

Chemotherapy as a conventional treatment aims for reduction, suppression, and removal of cancer, but cancer patients commonly suffer from severe side effects from it. Furthermore, in cancer patients, tumor-induced symptoms and chemotherapy-induced symptoms are often manifested in combination.^[1]^ Therefore, there has been an increasing demand for complementary and alternative medicines from both Korean medical doctors and patients to relieve cancer pain and side effects during the treatment. This will eventually improve the quality of life of cancer patients.^[2]^ The treatments based on traditional Korean medicine (TKM), a branch of complementary and alternative medicines, have been successfully applied to cancer patients to control the side effects of antineoplastic medicine in combined Oriental and Western medicine (COWM).^[3–4]^ Compared to Western medicine, TKM is fundamentally different in the treatment process for cancer patients; there is an important step of “pattern identification” for patient-specific diagnosis and medication. Pattern identification is a practice in TKM where the treatment is determined after the body constitution of the patients and their symptoms are holistically examined.^[5]^ These treatments are generally determined by the patients’ pattern identification as well as the diagnosed disease and are thus personalized medicine. Precision medicine, also known as personalized medicine, is an emerging treatment strategy targeted to the needs of individual patients on the basis of genetic, biomarker, phenotypic, or psychosocial characteristics that distinguish a given patient from other patients with similar clinical presentations.^[6–7]^ Therefore, Asia including Korea has already been using precision medicine according to patients’ pattern identification type traditionally, and TKM determined by pattern identification is a precision medicine by itself.

Pattern identification of each patient has not been widely applied to clinical trials in which objective criteria are important.^[8]^ But since clinical trials using pattern identification are gradually increasing as the importance of precision medicine is being recognized^[9]^, there has been studies using pattern identification in clinical trials for cancer patients.^[10]^ Also, traditionally diagnoses of pattern identification relied on patients’ subjective descriptions and doctors’ subjective judgments^[11–13]^, many studies on questionnaires such as body constitution questionnaire (BCQ) have been developed.^[14–17]^ This BCQ has been utilized to the clinical studies including breast cancer^[18]^, liver cancer^[19]^, diabetes^[20–22]^, schizophrenia^[23]^, and menopausal symptoms^[24]^, as well as healthy control groups^[25]^. BCQ enables us to assess patients’ pattern identification types according to their body constitutions using 19 to 23 simple-to-answer (survey) questions. This pattern identification tool can be more objective compared to the examination by individual TKM doctors.^[26–28]^ However, BCQ still requires a proven scientific basis for determining pattern identification of patients as well as wide acceptance from the TKM community. This study intends to find a scientific standard on the pattern identification of lung cancer patients in the framework of TKM.

Lung cancer was reported to be ranked first in both the number of new cases and the number of deaths for both genders among 36 types of cancers (excluding non-melanoma skin cancer) in 2018 which was estimated by The International Agency for Research on Cancer (IARC) to inform about cancer control and cancer research from 186 countries.^[29]^ For males, in particular, lung cancer also leads all other cancers in both the number of new cases and the number of deaths. It is notable that for both genders, the number of deaths from lung cancer is more than twice that of colorectum cancer, the second-tier in the number of deaths.^[30]^ In Korea, lung cancer is also the leading cause of death for both genders among the 24 types of cancers according to recent cancer statistics reports jointly published by Ministry for Health and Welfare and National Cancer Center of South Korea in 2018.^[31]^ These cancer statistics illustrate that lung cancer causes the largest number of deaths worldwide and indicate the importance of lung cancer therapy for cancer patients worldwide.

Cancer-associated glycoproteins have been reported to be shed into blood and lymph.^[32–33]^ Since glycans in glycoproteins are significantly altered in cancer^[34]^, they may serve as a tool for judging chemotherapy efficacy. Recent studies have reported changes in α-1-antitrypsin (A1AT) glycosylation in lung cancer serum, tissues, and cell lines.^[35–37]^ According to this study, a lectin microarray was used to detect glycosylation changes in serum A1AT from patients with lung adenocarcinoma (ADC), squamous cell lung cancer, small-cell lung cancer (SCLC), and benign pulmonary diseases. Differentially expressed glycosylated patterns of A1AT were identified by lectin arrays and were confirmed by lectin-based enzyme-linked immunosorbent assay (ELISA). This observational study is supported by these studies and suggests that the glycoproteomics approach may identify glycosylation differences among the 3 different pattern identification types in lung cancer plasma. Lung cancer patients will be pooled based on their pattern identification types diagnosed with BCQ assessment and then the glycoproteomics approach will be applied to each pooled pattern identification plasma.

## 2 Methods and analysis

### 2.1 Study design and period

**Figure 1:**
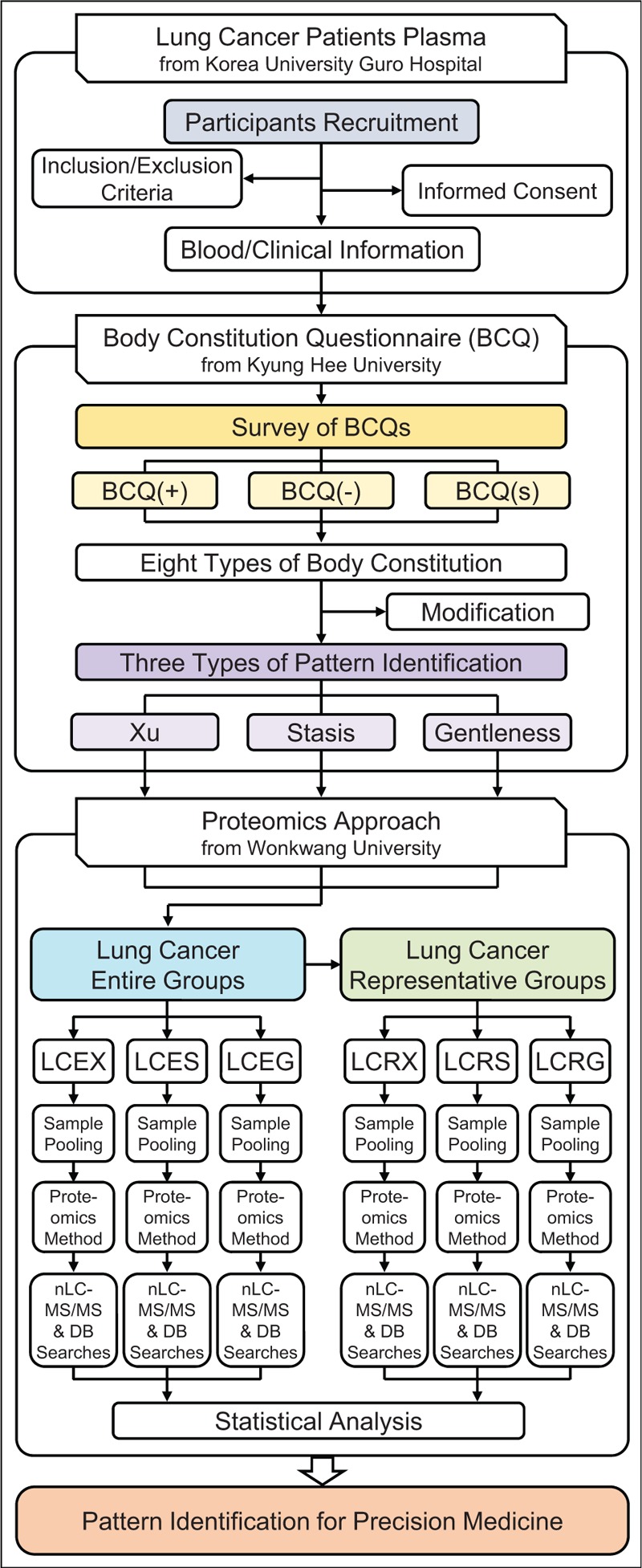
Flow chart of the experimental methodology.

This observational trial will at first diagnose the pattern identification types of lung cancer patients with BCQ and then elucidate their relationships with proteomics. The study will be carried out in 2 groups; a lung cancer entire (LCE) group and a lung cancer representative (LCR) group, and these 2 groups will be described in further sections in detail. Figure 1 shows the schematic flow of this study.

### 2.2 Outcomes

#### 2.2.1 Primary outcomes

1. Diagnosis of each pattern identification of Korean lung cancer patients with BCQs.
2. Verification of the correlation of BCQ and blood on each patients’ pattern identification type through glycoproteomics.
3. Protein identification and categorization by patients’ pattern identification for patient-specific diagnosis and medication in TKM.

#### 2.2.2 Long term outcomes

(1) Diagnosis of the pattern identification of Korean patients with scientific basis.
(2) Biomarker discovery on each pattern identification type of lung cancer patients within a large population.
(3) Development and commercialization of precision medicine on each pattern identification type.
(4) Tracking the efficacy of patient-specific treatments according to each pattern identification type with clinical proteomics in the follow-ups.

### 2.3 Recruitment

Recruitment of participants began in September 2015, and the participants are lung cancer patients from Korea University Guro Hospital (Seoul, Korea). All patients are Korean males and females over the age of 18 years. Participants are recruited by a lung cancer doctor in the University Guro Hospital (Seoul, Korea) if they fulfill the eligibility criteria recommended by the investigators.

### 2.4 Eligibility criteria

The inclusion and exclusion criteria of this trial are described in Table 1.

**Table 1:**
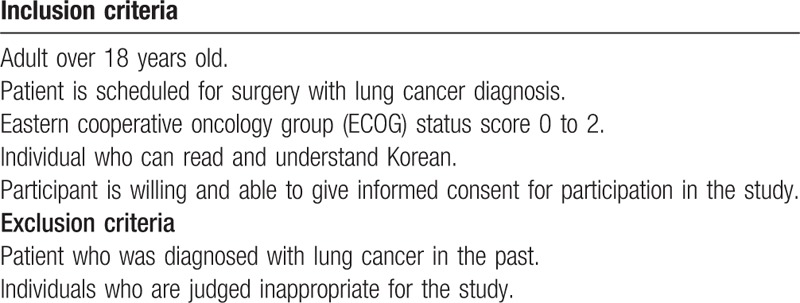
The inclusion and exclusion criteria.

### 2.5 Body constitution questionnaire (BCQ)

Pattern identification of a patient, expressed as either Yang or Yin, determines the patient's sensitivity for pathology. Yang deficiency (Yang-Xu) indicates a decrease of energy level in the physiological function of body and may cause fatigue, shortness of breath, chills, loose or watery stool, and polyuria.^[27]^ Yin deficiency (Yin-Xu) indicates a decrease in many physical aspects of human body including blood, interstitial fluid, and other bodily fluids and may result in thirsty, flush, dry stool, and decrease in urine output.^[26]^ Stasis is the inadequate transport of the physical aspects of the human body by energy due to the imbalance between Yin and Yang. Blood stasis and phlegm^[38]^ result from Stasis.^[28]^ Symptoms including joint pain, chest tightness or pain, lumps, and cough may arise. The BCQ(+), BCQ(−), and BCQ(s) questionnaires are developed in order to differentiate among Yang-Xu, Yin-Xu, and Stasis with a more objective method.^[26–28,39–41]^

#### 2.5.1 Initial classification

BCQ(+) is a questionnaire that is comprised of 19 questions with 1-to-5 rating scales, making the range of total score to be from 19 to 95 points with the cut-off score of 30.5 points for Yang-Xu.^[27]^ BCQ(−) is composed of another set of 19 questions with the same 5-point Likert scales, with the cut-off score of 29.5 points for Yin-Xu.^[26]^ BCQ(s) consists of 23 questions with the same Likert scale and cut-off score of 26.5 points for Stasis.^[28]^ A patient is diagnosed as Yang-Xu, Yin-Xu, and/or Stasis if his/her total score exceeds the corresponding cut-off score for each respective questionnaire. Therefore, patients will be categorized into 8 body constitutions of Yang-Xu, Yin-Xu&Yang-Xu, Yang-Xu & Stasis, Yin-Xu, Yin-Xu & Yang-Xu & Stasis, Yin-Xu & Stasis, Stasis, and Gentleness based on their BCQ scores.^[23,25]^

#### 2.5.2 Classification used in this study

Yang-Xu or Yin-Xu constitution is shaped by the persisting decrease of Yang or Yin, so patients who correspond to either Yang-Xu or Yin-Xu (not mutually exclusive) will be classified into Xu (Deficiency) group. On the other hand, patients that are diagnosed as Stasis through BCQ(s) will be included in Stasis group since “Stasis” constitution is formed by the persistent disruption of the dynamic balance between Yin and Yang.^[26–28]^ Patients who belong to none of the 3 body constitutions (Yang-Xu or Yin-Xu, Stasis) will be classified into the Gentleness group^[23,25]^. Therefore, the 8 body constitution types will be regrouped into 3 pattern identification types, Xu, Stasis, and Gentleness.

### 2.6 Lung cancer entire (LCE) groups

#### 2.6.1 Clinical information

Lung cancer patients will be interviewed face-to-face for 19 categories of clinical information, including sex, age, race, body weight, body height, disease history (cancer, diabetes, angina/myocardial infarction, and hypertension), surgical history, family history, medication history, smoking, alcohol, and cancer diagnostic information (diagnosis, surgery type, clinical TNM stage, pathological TNM stage, and cancer subtypes).

#### 2.6.2 Holistic viewpoint

Lung cancer patients will be randomly recruited without any prior categorization of their body constitutions. A disease of a patient with a certain type of pattern identification may be considered as arising from the patient's body constitution. Thus, it is important to holistically analyze all the lung cancer samples according to their pattern identification types.

#### 2.6.3 Sample groups on LCE groups

All the lung cancer patients will be divided into 3 LCE groups according to their pattern identification types (Xu, Stasis, or Gentleness). The patients who will be assessed as Xu will belong to lung cancer entire Xu (LCEX) group. Likewise, the patients who will be assessed as Stasis will be included to lung cancer entire Stasis (LCES) group, while patients to be assessed as Gentleness will belong to lung cancer entire Gentleness (LCEG) group.

### 2.7 Lung cancer representative (LCR) groups

#### 2.7.1 Definition of representative sample

Sampling is particularly crucial when heterogeneous materials are analyzed. It is critical that the analysis aims are clearly declared and that an appropriate sampling procedure is adopted. In some situations, a sampling strategy needs to be devised in order to optimize the value of the analytical information collected. A representative sample is what truly reflects the composition of the material to be analyzed within the context of a defined analytical problem.^[42]^

#### 2.7.2 Control factors

Glycoproteins in blood can vary qualitatively and quantitatively by numerous variables such as sex, age, and medical history. The glycoproteins must be qualitatively and quantitatively analyzed based on the pattern identification of the lung cancer patients. We hypothesize that each pattern identification of TKM as well as other variables such as sex, age, medical history, and etc. affects glycosylations on plasma proteins. Therefore, all the other variables other than pattern identification must be strictly controlled.

Since it is impossible to control all of the 19 variables of clinical information, we need to select as many variables as possible that are within our control. For example, smoking can be selected as the control factor that is closely related to lung cancer while sex ratio and average age can be considered as basic control factors for qualitative and quantitative analysis of glycoproteins. Both TNM stage and cancer subtype can be selected as control factors. Disease histories can be selected as control factors that can affect glycosylation in cancer plasma. Patients with no prior history of cancers other than lung cancer will be selected because other cancers may affect the aberration in the glycan moieties of glycoproteins in the plasma. Cancer histories from direct family can be considered as a genetic factor.

#### 2.7.3 Representative groups

The LCR groups will be generated from each LCE group by applying the selected control factors to lung cancer patient samples. The selected samples from LCEX group will be included in the lung cancer representative Xu (LCRX) group. Likewise, the selected samples from LCES group will be assigned to the lung cancer representative Stasis (LCRS) group while samples from LCEG group will be included in the lung cancer representative Gentleness (LCRG) group. The representative samples must be selected to have the same numbers of samples in each control factor for all pattern identification groups. It is also crucial to select a sufficient number of samples in order to reflect each pattern identification group. The number of patients with each control factor will be kept constant in each representative group.

#### 2.7.4 Scientific validation of entire groups using representative groups

Comparison of the identified glycoproteins between the LCE groups and the LCR groups with the same pattern identification type may reveal whether each LCR group truly represents each LCE group. This will be used to validate the unique glycoproteins from each pattern identification type of lung cancer patients under the framework of TKM.

### 2.8 Sample size

As a preliminary trial for exploring proteomics approach based on pattern identification, we intend to enroll 100 participants.

#### 2.8.1 LCE groups (LCEX, LCES, and LCEG)

At least 10 patients will be assigned to each group.

#### 2.8.2 LCR groups (LCRX, LCRS, and LCRG)

All LCR groups must have the same number of samples. The number of samples in these 3 LCR groups will be set by setting all the control factors other than pattern identification constant.

### 2.9 Samples preparation

Ten milliliters of blood will be withdrawn from each lung cancer patient before surgery and mixed on a blood mixer at room temperature. The mixed blood will be centrifuged for plasma separation from blood cells and the supernatant plasma will be transferred to a tube and stored in −80°C deep freezer until filtration. All the plasma samples will be filtered and pooled together according to each LCE group and LCR group. The 6 pooled samples will be stored in a −80°C deep freezer until affinity selection.

### 2.10 Glycoprotein capture

Glycoprotein capturing lectin column will be self-packed and connected to high performance liquid chromatography (HPLC) system for lectin affinity chromatography (LAC). Pooled human plasma from lung cancer patients will be loaded directly onto the column with mobile phase. The detailed affinity chromatographic method will follow the method section of the reference paper.^[43]^

### 2.11 Trypsin digestion and deglycosylation with PNGase F

The selected proteins will be digested with sequencing grade trypsin for protein digestion and then with PNGase F for deglycosylation of glycoproteins. The detailed proteomics method will follow the method section of the reference paper.^[44]^ The PNGase F-treated peptide mixtures will be stored at −80°C until analyses.

### 2.12 nLC-MS/MS methods for protein identification

The PNGase F-treated peptide mixtures from the 6 groups (LCEX, LCES, LCEG, LCRX, LCRS, and LCRG) will be analyzed with a nano liquid chromatography-tandem mass spectrometry (nLC-MS/MS). Each sample will be run more than twice under the identical conditions. The operational instruments and detailed methods will be in the method section of the reference paper.^[45]^

### 2.13 Statistical analysis with database searches

After at least 2 MS/MS data are obtained from each sample, protein identification will be performed with 2 complementary database search programs. Both Proteome Discoverer 1.4 (Thermo Fisher Scientific Inc., USA) and ProteinPilot 5.0 (SCIEX, USA) will be used to identify proteins from MS and MS/MS spectra. Each software program has its own unique analysis algorithm. Thus, it will be meaningful to compare the results from the 2 programs because it will reinforce the confidence of the identified proteins. At least 4 data files with identified proteins will be produced from each sample. Each data file will be individually sorted and filtered under the following conditions:

(1) dynamic modification option: deamidated (N-glycosylation)
(2) peptide confidence levels: the peptide is significant only if (a) confidence levels are over 95%, or (b) confidence levels are below 95% in 2 runs but show significant MS/MS spectra.
(3) proteins inclusion and exclusion: for Proteome Discoverer software, only the proteins with protein scores of 6.0 or higher will be included; for ProteinPilot software, only the proteins with the unused values of 2.0 or higher will be included. The rest of the identified proteins will be excluded from the results.

Starting with the significant data from the 2 software programs, only the peptides/proteins with N-glycosylation sites will be selected and used for the parent protein identification. These glycoproteins will be sorted and compared within each group (Xu, Stasis, and Gentleness). Then these proteins will be sorted into those that are identified in all LCE groups, all LCR groups, and only certain group(s). The resulting 6 N-glycoprotein lists will be used for the following 5 comparative analyses:

(1) the glycoprotein comparison sets from the first and second MS/MS runs for the confirmation of reproducibility
(2) the glycoprotein comparison sets from the 2 protein search engines to increase the confidence level of identified proteins
(3) the glycoprotein comparison sets between LCE groups and LCR groups to check the representativeness
(4) the unique and intersection glycoproteins set by comparison of LCE groups (LCEX, LCES, and LCEG) based on their pattern identifications assessed from BCQs of the lung cancer patients
(5) the unique and intersection glycoproteins set by comparison of LCR groups (LCRX, LCRS, and LCRG) based on their pattern identifications assessed from BCQs of the lung cancer patients.

**Figure 2:**
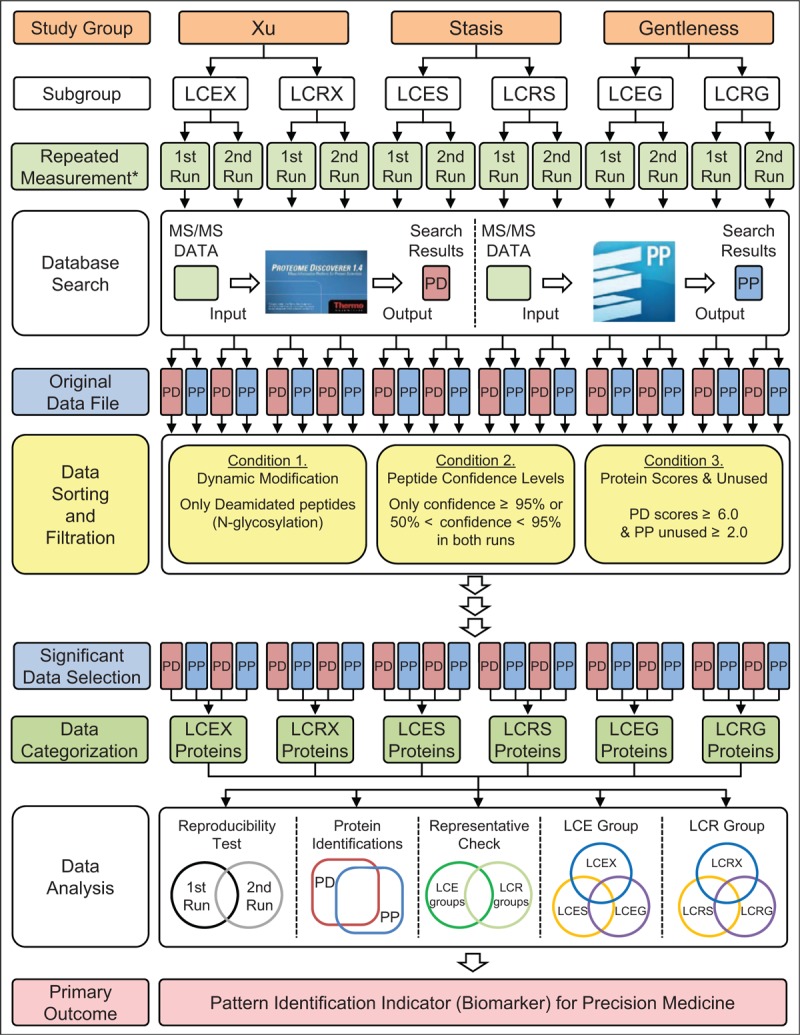
Flow chart for protein identification and classification using statistical analysis with database searches. ∗At least 2 MS/MS data will be obtained from each sample.

The overall data processing and analysis procedures are described in Figure 2. Unique glycoproteins from each group with each pattern identification of lung cancer patients will be generated from the results.

### 2.14 Ethics approval and consent to participate

The study was approved by the Institutional Review Board (IRB) of Kyung Hee University (Seoul, Korea) and Korea University Guro Hospital (Seoul, Korea). The reference numbers are KUGH15010–002 for Korea University Guro Hospital and KHSIRB-15–005(RA) for Kyung Hee University. Prior to undertaking any study-related procedures, written informed consent will be collected from all participants.

### 2.15 Monitoring of the study

Trial monitoring is performed by the Institute of Safety, Efficacy and Effectiveness Evaluation for Korean Medicine of the Kyung Hee University, Korea. A qualified investigator monitors this study. The investigator ensures that all the procedures are carried out, recorded, and reported in accordance with the standard operating procedures and all applicable regulatory requirements. Because this is a non-interventional study, it represents no risk or benefit for the patient.

A data monitoring committee (DMC) is not needed in this study since this is an observational trial and there are no intervention or security risks for patients.

## 3 Discussion

This is the first study in Korea to diagnose pattern identification with BCQ assessment which was proven to obtain validity and reliability in Taiwan^[39–40]^ and Hong Kong^[41]^. This study is also the first to apply BCQ assessment to lung cancer patients. Furthermore, this is the first to correlate BCQ and patients’ blood through glycoproteomics. After diagnosis of pattern identification type with BCQ, glycoproteomics approach will be introduced to all the plasma in order to establish scientific and biomedical criteria on BCQ for the first time. Two sets of patients’ plasma will be explored from each pattern identification type of TKM; a holistic evaluation (LCE groups) and a scientific validation of the representativeness (LCR groups). It is regarded that the diseases of a patient with specific pattern identification arise from the corresponding pattern identification. Thus, it is important to holistically analyze the entire lung cancer patients according to their pattern identification types. On the other hand, by carefully selecting appropriate control factors, all the control factors other than the pattern identification are prevented from interfering with the change in glycoproteins in their blood. This change may vary depending on diverse variables such as sex, age, and medical history.

The patient-optimized TKM for lung cancer can be applied to cancer therapy for future clinical trials and treatments by presenting scientifically valid criteria on patient's pattern identification from this trial. The identified plasma proteins from each pattern identification type will be categorized as pattern identification indicators (biomarkers) for precision medicine. In the long run, these proteins can also be utilized as lung cancer biomarkers aiming for development of in vitro lung cancer diagnostic kits based on pattern identification of lung cancer patients.

## Author contributions

WC has written this manuscript, is in charge of proteomics, and is one of the general supervisors for this research. JHK contributed to the design of this study, collected the specimens (coordinated to acquire/collect the specimens), and participated in the drafting the manuscript. MJ and JL contributed to the conception and design of proteomics approach and made a substantial contribution to the drafting of the manuscript. CC and SP contributed to the design of this study, to analyze the BCQ questionnaire and classification, and helped to draft the manuscript. MK and HS contributed to the collection of the specimens. SGK contributed to the funding, is one of the general supervisors for this research and participated in both the study design and critical revision of the manuscript. All authors read and approved the final manuscript to be submitted.

**Conceptualization:** Wonryeon Cho, Ji Hye Kim, Chunhoo Cheon, Sunju Park.

**Funding acquisition:** Seong-Gyu Ko.

**Investigation:** Chunhoo Cheon, Sunju Park.

**Methodology:** Miseon Jeong, Jinwook Lee.

**Project administration:** Wonryeon Cho, Seong-Gyu Ko.

**Resources:** Ji Hye Kim, Myeong-Sun Kim, Hyoungwoo Son.

**Supervision:** Wonryeon Cho, Seong-Gyu Ko.

**Writing – original draft:** Wonryeon Cho, Miseon Jeong, Jinwook Lee, Chunhoo Cheon.

**Writing – review & editing:** Wonryeon Cho, Ji Hye Kim.

Wonryeon Cho orcid: 0000-0002-9953-6176.
